# Inhibition of Adenylyl Cyclase Type 5 Increases Longevity and Healthful Aging through Oxidative Stress Protection

**DOI:** 10.1155/2015/250310

**Published:** 2015-04-07

**Authors:** Stephen F. Vatner, Ronald E. Pachon, Dorothy E. Vatner

**Affiliations:** Departments of Cell Biology & Molecular Medicine and Medicine, New Jersey Medical School, Rutgers University, Newark, NJ 07103, USA

## Abstract

Mice with disruption of adenylyl cyclase type 5 (AC5 knockout, KO) live a third longer than littermates. The mechanism, in part, involves the MEK/ERK pathway, which in turn is related to protection against oxidative stress. The AC5 KO model also protects against diabetes, obesity, and the cardiomyopathy induced by aging, diabetes, and cardiac stress and also demonstrates improved exercise capacity. All of these salutary features are also mediated, in part, by oxidative stress protection. For example, chronic beta adrenergic receptor stimulation induced cardiomyopathy was rescued by AC5 KO. Conversely, in AC5 transgenic (Tg) mice, where AC5 is overexpressed in the heart, the cardiomyopathy was exacerbated and was rescued by enhancing oxidative stress resistance. Thus, the AC5 KO model, which resists oxidative stress, is uniquely designed for clinical translation, since it not only increases longevity and exercise, but also protects against diabetes, obesity, and cardiomyopathy. Importantly, inhibition of AC5's action to prolong longevity and enhance healthful aging, as well as its mechanism through resistance to oxidative stress, is unique among all of the nine AC isoforms.

## 1. Introduction

Adenylyl cyclase (AC) is a ubiquitous enzyme which regulates all organs and catalyzes the conversion of ATP to cAMP. There are nine major mammalian AC isoforms; types 5 and 6 are the major isoforms in the heart. Since our laboratory has been primarily involved in cardiovascular regulation, our interest was in disrupting one of the two major isoforms in the heart, type 5 AC (AC5 knock out (KO)), to see how regulation of cardiac function is altered. We found that the AC5 KO heart was protected against the stresses of chronic pressure overload [1] and chronic catecholamine stimulation [2, 3]. However, even more interesting and potentially important were our other findings of the salutary effects of AC5 inhibition, not necessarily related to the heart. We also studied these mice for 3 years and found that they lived a third longer than wild type (WT) [4]. Although longevity models are important, the translational value for these studies is limited, unless the model also improves healthful aging. In fact older individuals often are not interested in expanding life span, if it is accompanied by many of the limitations observed in the elderly, for example, exercise intolerance, diabetes, and obesity. Importantly the AC5 KO model also promotes healthful aging, as it enhances exercise capacity, and protects against diabetes and obesity and diabetic cardiomyopathy [5]. Indeed the AC5 KO model shares many of the same features of the most commonly studied model of longevity and protection against obesity, caloric restriction [6]. However, caloric restriction does not improve exercise performance, but, on the contrary, it actually diminishes exercise capacity [7]. There is also one major limitation to the translation of caloric restriction to longevity and protection against diabetes and obesity in patients, that is, compliance due to difficulty in maintaining low calorie or low fat diets. Accordingly, there is considerable need for a novel therapy that might mimic the phenotype of caloric restriction, without the negative aspects of limiting caloric intake. One such model is inhibition of AC5, which has a similar phenotype to caloric restriction [6], but the mice actually eat more than their wild type controls, while weighing less.

One common mechanism that mediates longevity and healthful aging is protection against oxidative stress [4, 6, 8–22].  Table 1 summarizes the most commonly studied longevity models, showing that the majority of these models, as well as the AC5 KO model, have one common theme, that is, protection against oxidative stress. Accordingly, in order to understand the mechanisms mediating the beneficial effects of the AC5 KO model, it is important to examine its protection against oxidative stress.

## 2. Oxidative Stress in Aging and in the AC5 KO (Figures 1 and 2)

**Figure 1:**
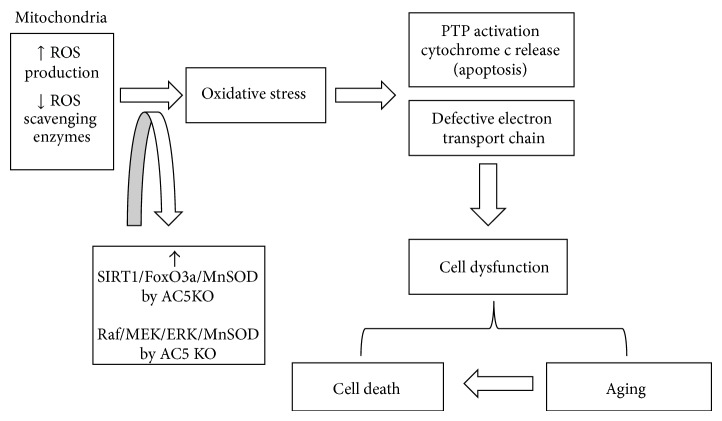
Mitochondrial dysfunction and oxidative stress. During the aging process, ROS accumulation and DNA mutation in mitochondria induce oxidative stress, which results in the mitochondrial permeability transition pore (PTP) opening and electron transport chain deficiency, thereby leading to cell dysfunction and cell death. Inhibition of AC5 activates SIRT1/FoxO3a and Raf/MEK/ERK pathways, and both pathways upregulate the antioxidant, MnSOD, resulting in resistance to oxidative stress during aging.

**Figure 2:**
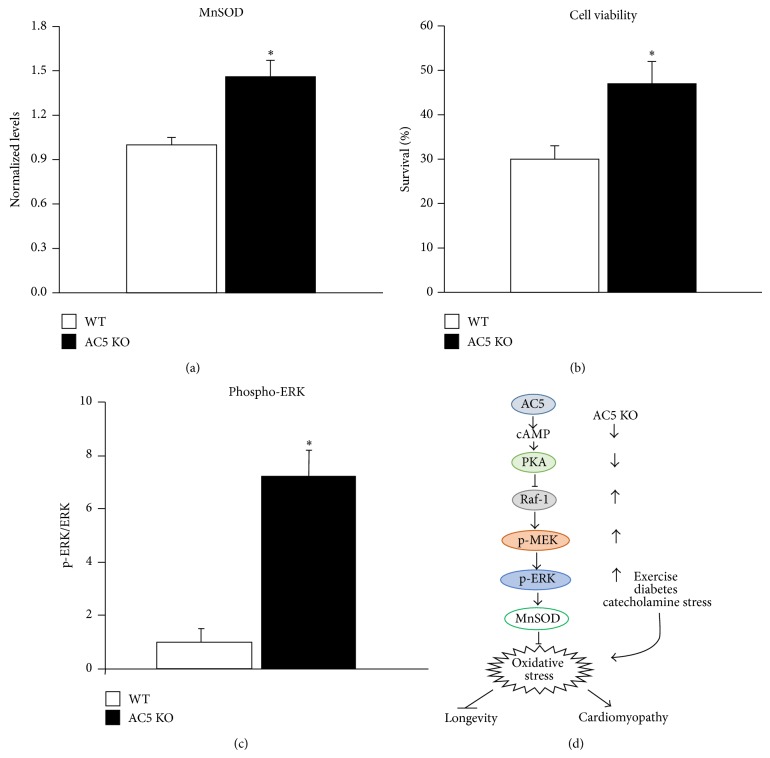
ERK pathway and oxidative stress in aging AC5 KO. (a) Western blotting of MnSOD in the hearts of aging WT and AC5 KO mice. The levels of MnSOD are significantly greater in AC5 KO mice compared to WT mice (^∗^ *P* < 0.05). Data are expressed as mean ± SEM. (b) Cell viability was tested in response to oxidative stress in neonatal cardiac myocytes from AC5 KO and WT. Myocardial cells were treated with H_2_O_2_ (25 *μ*M) and evaluated for cell viability using Cell Titer-Blue Cell Viability Assay. AC5 neonatal myocytes showed resistance to oxidative stress and DNA damage (^∗^ *P* < 0.05 versus WT). Data are presented as mean ± SEM. (c) By western blotting, the level of ERK phosphorylation was significantly increased in AC5 KO mice compared with WT (^∗^ *P* < 0.05). Data are expressed as mean ± SEM. (d) Signaling diagram for AC5 regulation of oxidative stress through the ERK pathway is shown. Data is redrawn from Yan et al. [4].

The AC5 KO model of aging protects against oxidative stress by reducing cAMP and protein kinase A (PKA), which in turn activates the Raf/MEK/ERK pathway, which increases MnSOD and protects against oxidative stress [4] (Figures 1 and 2). As noted above, protection against oxidative stress is a common mechanism in longevity models (Table 1). Oxidative stress is enhanced during the aging process; mitochondria produce less energy (ATP) and also increase the production of reactive oxygen species (ROS) as a product of aerobic metabolism (Figure 1). Furthermore the activity of free-radical scavenging enzymes changes with aging. One mechanism by which oxidative stress is increased in the aging tissue is through increased ROS induced apoptosis and necrosis by opening of the mitochondrial membrane permeability transition pore and release of apoptotic inducing factors, for example, cytochrome c [23] (Figure 1). Oxidative stress has been linked to aging and senescence through the tumor-suppressor p53 and transcriptional responses, mediated by p44/p53 and p66 [24]. There is also considerable support for the regulation of oxidative stress by the Raf/MEK/ERK pathway [25]. In summary, the mechanism of oxidative stress induced aging seems to be multifactorial, where mitochondrial damage and impaired function play a significant role. The final common pathway for premature cell death has been reported to be apoptosis, necrosis, and control of the cell cycle [26, 27] (Figure 1), specifically an increase in the percentage of cells stopped at the G0/G1 phase of the cell cycle [26].

To further understand the importance of oxidative stress in aging, the extent to which the major diseases limit longevity must be examined for their relation to oxidative stress. Based on the statistics of the CDC in 2010, the leading causes of death in humans in order of frequency are heart disease, cancer, chronic lower respiratory diseases, stroke, accidents, Alzheimer's disease, diabetes, influenza and pneumonia, nephritis, nephrotic syndrome, and nephrosis. It is important to recognize that most of these diseases are related to oxidative stress. Such is the case for heart disease [67], cancer [68], COPD [69], stroke [70], Alzheimer's [71], diabetes [72], and chronic kidney disease [73]. In mice, the causes of mortality also share oxidative stress mechanisms. The most common cause of death in C57BL/6J mice is neoplasia, including lymphoma and other hematological and nonhematological cancers, followed by chronic kidney and heart diseases [74]. As previously described, these entities have been linked to oxidative stress, further supporting the importance of oxidative stress in limiting longevity.

Perhaps the most convincing evidence for oxidative stress in aging is Progeria, a unique medical condition characterized by premature aging [75], such that teenagers often suffer from atherosclerosis, cardiomyopathy, and coronary artery disease and death. Interestingly, accumulation of oxidized proteins causing DNA damage has been described as the causative effect in this disease [76, 77]. One abnormally formed protein is called lamin A. It is an autosomal dominant mutation involving (LMNA) gene and/or abnormal posttranslational processing (ZMPSTE24) [75, 78].

## 3. Oxidative Stress in Exercise

The AC5 KO model is one of the few aging models known to also improve exercise performance [5]. This is important for two reasons. First, maintained exercise performance is common to longevity, as reduced exercise performance is common to many of the diseases that limit longevity, for example, cardiopulmonary diseases. Secondly, exercise training and conditioning is recognized to be therapeutic to most diseases and is one intervention that can extend longevity [79]. It is therefore important and well established that both resting and contracting skeletal muscles produce ROS. Exercise has been also related to induction of oxidative stress when it is performed at high intensity. However, moderate intensity aerobic exercise enhances endothelium dependent vasodilation through the increased production of nitric oxide [80]. This could be explained by the fact that while high levels of free radicals can damage cellular components, low to moderate level of oxidants play multiple regulatory roles in cells such as the control of gene expression, regulation of cell signaling pathways, and modulation of skeletal muscle force production [80].

There are multiple cellular mechanisms mediating resistance to oxidative stress, by regular moderate exercise. These include reduction of basal formation of oxidants, improvement of the antioxidant defense system, and increased resistance of tissues against ROS damage [81]. Furthermore, in a study using rats, exercise increased total serum antioxidant substances with an additional beneficial effect on lipid profile [82]. More specifically, another study evaluating the relationship of oxidative stress, endothelial dysfunction, and atherosclerosis with physical inactivity in mice showed that decreased physical activity increases vascular NADPH oxidase activity and enhances vascular ROS production, which contributes to endothelial dysfunction and atherosclerosis as opposed to physically active animals [83].

## 4. Oxidative Stress in Diabetes and Obesity

As noted above the AC5 KO mouse shares a common phenotype and genotype with caloric restriction [6], a frequently studied model demonstrating protection against diabetes and aging. The AC5 KO protection against glucose intolerance and insulin resistance and obesity is observed in the animals on a standard diet, but it is even more pronounced when stressed with a high fat diet [84]. The AC5 KO mice weigh less and have less obesity and better serum lipids as well as glucose tolerance and insulin resistance compared to the WT mice [84]. Glucose intolerance and reduced insulin sensitivity have also been linked to oxidative stress mechanisms. Chronic hyperglycemia leads to generation of ROS resulting in increased oxidative stress and destruction of pancreatic cells, critical to insulin secretion [72].

The AC5 KO is also protected from obesity [6], which is also linked to oxidative stress mechanisms. There are several mechanisms by which obesity produces oxidative stress. First, adipose tissue produces certain bioactive substances called adipokines such as IL-6 and leptin, which induce the production of ROS. A second mechanism is that mitochondrial and peroxisomal oxidation of fatty acids can produce ROS in oxidation reactions. And third, there is an overconsumption of oxygen in the mitochondrial respiratory chain [85]. Additionally, the pattern of food consumption in obese patients can potentially exacerbate the cellular damage; lipid rich diets also produce ROS due to modifications in oxygen metabolism contributing to the cell dysfunction induced by obesity [85]. Interestingly, the persistence of obesity in humans has been shown to decrease the activity of antioxidant enzymes in the adipose tissue such as catalase, superoxide dismutase, and glutathionine peroxidase [85, 86]. This could represent another mechanism involved in the progression of the disease and the development of obesity related complications. Finally, a study in humans confirmed the increased oxidative stress state seen in patients with obesity and diabetes, measuring urinary creatinine-8-epi-PGF2*α* as a marker of systemic oxidative stress [87]. Taking all the previous evidence together, it is clear that oxidative stress is a common factor in obese and diabetic patients.

## 5. Oxidative Stress in Cardiomyopathy and Heart Failure

There is accumulating evidence that increased oxidative stress is involved in the pathogenesis of various types of cardiomyopathy [88], including dilated [89, 90], diabetic [91, 92], ischemic [93, 94], hypertensive [95], adriamycin-induced [96], and pressure overload-induced cardiomyopathy [97–99], as well as beta adrenergic receptor overexpression induced cardiomyopathy [3, 100]. It is important to note that oxidative stress affects different cell types involved in cardiomyopathy, not just cardiac myocytes. Dysfunction in other cells, such as endothelial cells and fibroblasts, plays an important role in the development of cardiomyopathies, as well.

**Figure 3:**
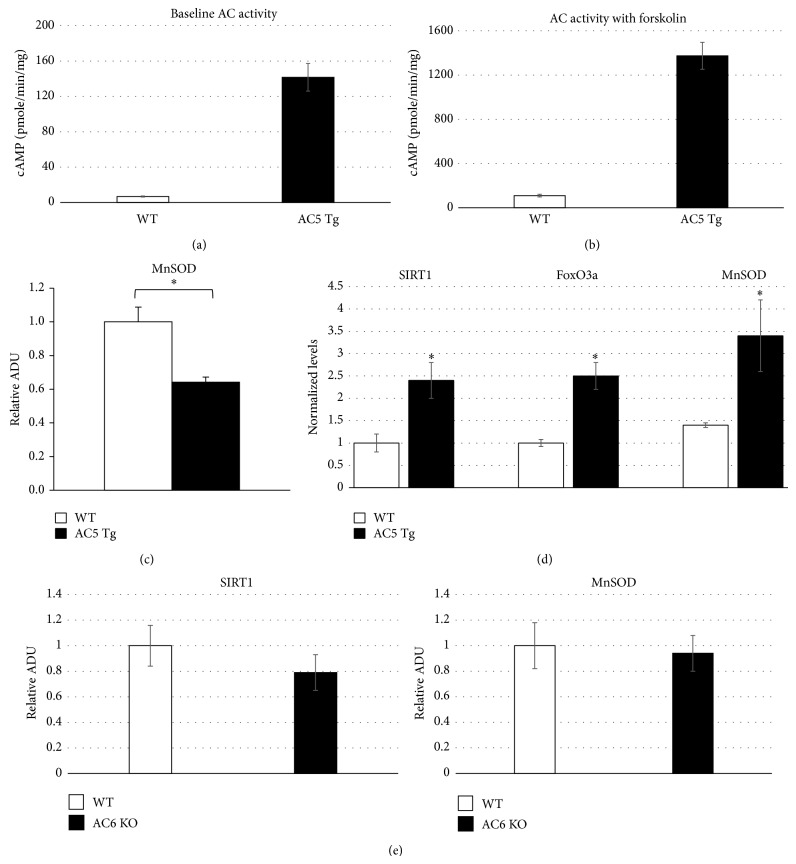
SIRT1/FoxO3a/MnSOD pathway in AC5 KO, AC5 Tg, and AC6 KO. (a) and (b) AC activity at baseline and in response to forskolin stimulation was enhanced in AC5 Tg mice compared to WT. (c) AC5 regulated MnSOD expression. Downregulation of MnSOD in AC5 Tg mice hearts is shown (*n*: 6 per group) (^∗^ *P* < 0.05). (d) Expression of SIRT1 by western blotting in the AC5 myocardial cells. SIRT1 was highly expressed compared to WT. (^∗^ *P* < 0.05). Expression of FoxO3a by western blotting in AC5 KO mouse hearts is shown. More FoxO3a was expressed in the nucleus of AC5 KO myocytes compared to WT. MnSOD expression increased in AC5 KO hearts (^∗^ *P* < 0.05). Data are expressed as mean ± SEM. (e) SIRT1 and MnSOD expression levels in AC6 KO did not show any difference compared to WT. Data is redrawn from Lai et al. [3].

**Figure 4:**
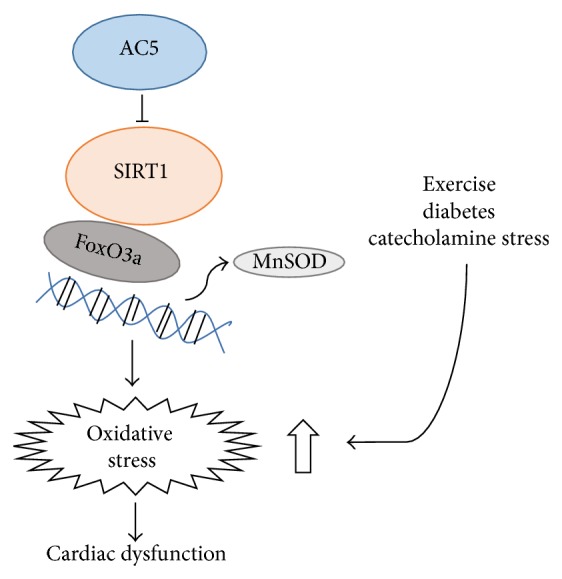
Signaling diagram for AC5 regulation of oxidative stress through the SIRT1/FoxO3a pathway.

**Figure 5:**
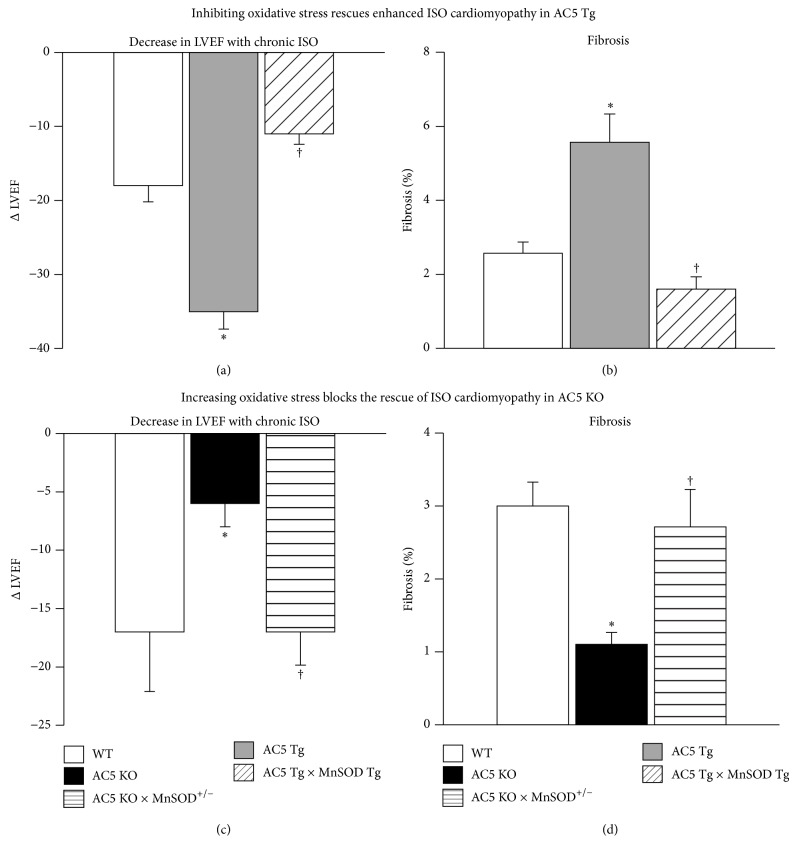
The effects of chronic isoproterenol (ISO) on AC5 Tg hearts ((a) and (b)). Chronic ISO exacerbated cardiomyopathy in AC5 Tg compared with WT, as reflected by a greater decrease in left ventricular ejection fraction (LVEF) (a) and more fibrosis (b) (^∗^ *P* < 0.05). Mating the AC5 Tg mice with MnSOD Tg (AC5 Tg × MnSOD Tg) mice rescued ISO cardiomyopathy. The effects of chronic ISO on AC5 KO hearts are shown ((c) and (d)). Chronic ISO reduced cardiomyopathy in AC5 KO compared with WT, as reflected by less of a decrease in LVEF (a) and less fibrosis (b). Mating the AC5 KO mice with MnSOD heterozygous mice (AC5 KO × MnSOD^+/−^) eliminated the protective effects of AC5 KO with chronic ISO. ISO: isoproterenol. ^∗^ *P* < 0.05. Data is redrawn from Lai et al. [3].

The AC5 KO model is also protected against cardiomyopathy and heart failure through oxidative stress mechanisms [3] (Figures 3–5). For example, chronic beta adrenergic receptor (*β*-AR) stimulation induces cardiomyopathy and heart failure by increasing markers of oxidative stress damage including myocyte necrosis and apoptosis [100]. We found that augmenting oxidative stress by mating the AC5 KO mice with MnSOD KO mice resulted in loss of the protection against the decreased cardiac function and increased cardiac fibrosis in response to chronic catecholamine stimulation in the double knockouts (Figure 5) [3, 5]. Conversely, when AC5 is overexpressed in the heart, as occurs in the cardiac specific AC5 Tg mouse, the cardiomyopathy induced by chronic catecholamine stimulation is exacerbated (Figure 5). In this model mating the AC5 Tg mice with MnSOD Tg mice rescues the cardiomyopathy (Figure 5). Furthermore, AC5 KO can also prevent the cardiomyopathy induced by chronically enhanced *β*-AR signaling in mice with overexpressed *β*2-AR also, potentially, through enhancing resistance to oxidative stress [100]. These findings confirm the importance of oxidative stress in the pathogenesis of heart failure in general and in the protection afforded by the AC5 KO model in particular. The AC5 KO model is also protected against cardiomyopathies induced by chronic pressure overload [1], diabetes [5], and aging [4].

## 6. Mechanisms of AC5 KO Induced Longevity and Oxidative Stress Resistance

### 6.1. ERK Signaling Pathway Related to AC5 KO and Oxidative Stress (Figure 2)

We previously found that AC5 KO increases longevity and stress resistance via activation of the Raf/MEK/ERK signaling pathway [4]. This finding, based on the reduction in cAMP by AC5, is supported by studies showing that the AC/cAMP/PKA pathway negatively regulates the MEK-ERK signaling pathway [101]. The MEK/ERK signaling pathway is also one of the main stress signaling pathways and central mediators activated in response to oxidative damage [102, 103]. Previous findings suggested that a decrease in the activation of the Raf/MEK/ERK pathway is associated with aging [104–109]. For example, decreased levels of ERK phosphorylation were observed in senescent fibroblasts [104, 110] and hepatocytes from aging rats [111], whereas caloric restriction, a well-recognized mechanism mediating longevity and stress resistance, significantly reduced the age-related decline in ERK activation [111]. The long-lived Snell dwarf mice also exhibits an elevated level of ERK phosphorylation [112]. We have shown an activation of the MEK/ERK signaling pathway in various tissues from long-lived AC-5 KO mice, which is consistent with that found in caloric restricted mice and long-lived Snell dwarf mice. Interestingly, recent studies demonstrated a slower and more prolonged activation of ERK with longevity [113]. Furthermore, the activation of the ERK pathway in response to oxidative stress is reduced with age, while loss of oxidative stress resistance with aging is associated with decreased ERK activity, implying that ERK activation exerts a prosurvival signal against aging induced oxidative stress [111].

In addition, superoxide dismutase (SOD) has been reported as the downstream target of the Cyr1 (AC)/cAMP/PKA pathway in yeast, which induces protection [114]. We have shown that MnSOD, which is a major molecule protecting against oxidative stress, is upregulated in AC5 KO mice (Figure 2(a)) [4] but downregulated, when AC5 is upregulated, as in the AC5 Tg heart (Figure 3(c)) [3]. A deficiency of SOD is able to induce senescence; for example, homozygous SOD2−/− mice show significant damage to mitochondrial DNA in lung and liver compared to WT mice or heterozygous mice, resulting in a survival rate of only up to seven days after birth due to cardiomyopathy and liver disease [115]. The linkage between SOD and ERK activation is controversial, however. Although EGF-induced phosphorylation of ERK1/2 is attenuated by overexpression of Cu/ZnSOD in vascular smooth muscle cells, several other studies have shown that the ERK signaling cascade plays a positive role in overexpression of MnSOD, which protects murine fibrosarcoma cells from apoptosis [116] and suppresses tumor growth in the mac25/IGFBP-rP1-transfected human breast and prostate cancer cell lines [117]. Our findings have proven that MnSOD is the downstream enzyme involved in the ERK signaling cascades [3, 4] mediating longevity and stress resistance in AC5 KO mice.

### 6.2. AC5, SIRT1, FoxO3a, and MnSOD (Figures 3 and 4)

As noted above, we found that AC5 KO increases life span and protects against oxidative stress though upregulating the antioxidant, MnSOD [4], whereas MnSOD regulated the cardiomyopathy induced by chronic catecholamine stimulation through the AC5, SIRT1, FoxO3a, and MnSOD pathway [3]. MnSOD is regulated transcriptionally by several transcription factors, such as NF-*κ*B, p53, and FoxO3a [118–120]. Among them, FoxO3a is most closely related to the antiaging effects of MnSOD. It is known that FoxO3a is essential for the participation of MnSOD in antiaging mechanisms of various species. In* C. elegans*, FoxO3a transcriptionally upregulated MnSOD, which induced life span extension [121]. In rats, aging induced downregulation of MnSOD is due to the inactivation of FoxO3a [122]. In human quiescent cells, FoxO3a binds directly to the promoter of MnSOD and protects the cells from oxidative stress [123]. The transcriptional activity of FoxO factor could be activated by deacetylation. SIRT1 is a deacetylase which is able to activate FoxO3a (Figure 4) [118]. Interestingly, we found that the SIRT1/FoxO3/MnSOD pathway is only activated by AC5 and not by AC6 (another major AC isoform in the heart and brain), indicating a unique regulation of this pathway by AC5 (Figure 3(e)) [3]. The Puigserver lab reported a new short-term SIRT1 activation pathway that involved *β*-AR/AC/cAMP/PKA [124]. However, the AC5 KO model is quite different, since it increases NAD^+^ content and SIRT1 protein expression, which chronically maintains NAD^+^/SIRT1 levels and mitochondrial activity to meet energy requirements. Importantly, the AC5/SIRT1 regulation is unique to AC5, since our data show that AC5 KO is resistant to obesity [6], but AC6 KO did not affect body weight and SIRT1 expression [3] (Figure 3), and AC3 KO actually induced obesity [125].

### 6.3. AC5 Is Unique among the 9 AC Isoforms (Table 2)

It is interesting that there are 9 major mammalian isoforms of AC and many share regulation of the same tissue or organ in the body [126, 127]; yet they regulate in radically different ways. One example relates to the two major isoforms of AC in the heart, AC5 and AC6. Inhibiting AC6 shares none of the salutary features of inhibiting AC5, as summarized in this review, with one exception; that is, there are two divergent reports on the regulation of cardiomyopathy by the AC6 KO model: one which claims it is protective [128], as we demonstrated for the AC5 KO [1, 2], and another with the opposite conclusions; that is, AC6 KO results in more severe cardiomyopathy [129]. The results for cardiac specific AC 6 transgenic models are also controversial, with some demonstrating protection [130–133] and another showing the reverse [134]. It is even more interesting and pertinent to the topic of oxidative stress that so little is known about AC isoforms and regulation of oxidative stress. Almost none of the other 8 AC isoforms have been shown to regulate oxidative stress (Table 2), with the tangential exception that AC1 can affect glutamate induced toxicity, which is related to oxidative stress, in cortical neurons. Although little is known about regulation of oxidative stress by the other AC isoforms, the reverse has been shown, that is, oxidative stress regulation of AC isoforms [135, 136]. In addition, the major mechanisms mediating oxidative stress in the AC5 KO, the SIRT1/FoxO3/MnSOD pathway, have also not been observed with the other AC isoforms (Table 2). It could well be that other AC isoforms do regulate oxidative stress but that this just has not been studied as of yet and would be an important future direction for AC research.

## 7. Summary

It has been recognized for some time that protection against oxidative stress is a common mechanism mediating longevity (Table 1). This mechanism is also critical in understanding why inhibition of AC5, as in the AC5 KO model, extends longevity. But more importantly, the lesson from the AC5 KO model is how oxidative stress is important in mediating healthful aging, which when coupled to longevity provides a blueprint for clinical translation. Inhibition of AC5 also protects against diabetes and obesity and cardiomyopathy, while improving exercise performance. Resistance to oxidative stress plays a role in mediating all of these salutary features of the AC5 KO. Importantly, AC5 is the only one of the 9 AC isoforms to demonstrate longevity and healthful aging through resistance to oxidative stress.

## 8. Clinical Translation

Since inhibition of AC5 extends longevity and protects against diabetes, obesity, and cardiomyopathy, while improving exercise tolerance, it naturally becomes an important mechanism for clinical translation. There have been recent clinical studies supporting our findings in the AC5 KO model. The clinical genome wide association studies have identified single nucleotide polymorphisms (SNPs) in the ADCY5 gene associated with increased type 2 diabetes risk [137], which is the inverse of AC5 inhibition and therefore consistent with our findings. However, it is difficult to isolate the specific action of one gene in human genome studies, as we have done by disrupting the AC5 gene in mice. Unfortunately disrupting the AC5 gene in patients is not feasible and therefore it becomes necessary to identify a pharmacological inhibitor of AC5. One example of a pharmacological compound that replicates many of the features of AC5 inhibition is an FDA approved antiviral drug, Vidarabine [138], which protects against the development of cardiomyopathy in mice [139]. However, this drug is not purely an AC5 inhibitor and has the disadvantage that it cannot be administered orally. Accordingly, further work is required to develop a nontoxic AC5 inhibitor that is soluble and can be given to patients orally.

## Figures and Tables

**Table 1:** Longevity and oxidative stress resistance.

Longevity models related to oxidative stress resistance	Longevity models not related to oxidative stress resistance	Longevity models with relationship to oxidative stress resistance controversial or not studied
AC5 KO [3–6]	Gpx4 +/− KO [28]	Ames dwarf [29–31]
Caloric restriction [8, 32–35]	GHR/BP KO [36, 37]	FIRKO [38, 39]
Snell dwarf [40–42]	MIF KO [43, 44]	PAPPA^−/−^ KO [45, 46]
GHR KO [9, 40]	*α* MUPA OE [47, 48]	RII*β*−/− KO [49]
Igf-1r^+/−^ KO [10, 50, 51]		S6K1−/− KO [52, 53]
Klotho OE [11, 12, 54]		Hct-UCP2 OE [55]
p66^shc−/−^ [13, 56]		PEPCK-C OE [57]
TRX OE [14, 58]		PtenTg [59]
MCAT [15, 60–62]		SIRT6 Tg [63]
MT OE [16]		
Agtr1 *α*−/− KO [17]		
Irs1−/− KO [18]		
R6/2 Irs2+/− IRS2 Btg KO [19]		
Surf1−/− KO [21, 22, 64]		
Mclk1+/− KO [65, 66]		

Data are shown as mice models followed by the supporting bibliography in parenthesis [].

**Table 2:** Adenylyl cyclase (AC) isoforms, which protect against oxidative stress using the SIRT1/FoxO3/MnSOD pathway.

	AC 1	AC 2	AC 3	AC 4	AC 5	AC 6	AC 7	AC 8	AC 9
Oxidative stress protection	+	?	?	?	+	?	?	?	?
SIRT 1	?	?	?	?	+	−	?	?	?
FoxO3	?	?	?	?	+	?	?	?	?
MnSOD	?	?	?	?	+	−	?	?	?

+ Positive, − negative, and ? unknown.
